# A rapid review of antenatal hepatitis C virus testing in the United Kingdom

**DOI:** 10.1186/s12884-023-06127-x

**Published:** 2023-11-28

**Authors:** M. P. Hibbert, R. Simmons, S. Mandal, C. A. Sabin, M. Desai

**Affiliations:** 1 Sexually Transmitted Infections and HIV Division, Blood Safety, Health Security Agency (UKHSA), Hepatitis, London, England, UK; 2 National Institute for Health and Care Research Health Protection Research Unit (NIHR HPRU) in Blood Borne and Sexually Transmitted, Infections at University College London in partnership with UKHSA, London, England; 3 Institute for Global Health, University College London, London, England

**Keywords:** Hepatitis C virus, Antenatal services, Rapid review

## Abstract

**Background:**

The United Kingdom (UK) has committed to the World Health Organization’s viral hepatitis elimination targets. New case finding strategies, such as antenatal testing, may be needed to achieve these targets. We conducted a rapid review to understand hepatitis C-specific antibody (anti-HCV) and HCV RNA test positivity in antenatal settings in the United Kingdom to inform guidance.

**Methods:**

Articles and conference abstracts published between January 2000 and June 2022 reporting anti-HCV testing in antenatal settings were identified through PubMed and Web of Science searches. Results were synthesised using a narrative approach.

**Results:**

The search identified 2,011 publications; 10 studies were included in the final synthesis. Seven studies used anonymous testing methods and three studies used universal opt-out testing. Anti-HCV test positivity ranged from 0.1 to 0.99%, with a median value of 0.38%. Five studies reported HCV RNA positivity, which ranged from 0.1 to 0.57% of the testing population, with a median value of 0.22%. One study reported cost effectiveness of HCV and found it to be cost effective at £9,139 per quality adjusted life years.

**Conclusion:**

The relative contribution of universal opt-out antenatal testing for HCV should be reconsidered, as antenatal testing could play an important role in new case-finding and aid achieving elimination targets.

## Background

 The World Health Organization’s (WHO) global health sector strategy aims to support the elimination of viral hepatitis as a public health threat by the year 2030 [1]. To eliminate viral hepatitis as a public health threat, new methods are needed to reduce incidence, morbidity and mortality from viral hepatitis. It is estimated that 95% of deaths from viral hepatitis are attributable to hepatitis B (HBV) and C (HCV) viruses and these are thus the primary focus of elimination [1]. HCV has a long progression time, remaining asymptomatic and often undetected for many years before liver cirrhosis may develop [2]. Internationally, there are approximately 290,000 deaths from HCV and 1.5 million new infections annually [3]. The United Kingdom (UK) has committed to the WHO strategy to eliminate viral hepatitis and England has achieved the WHO interim elimination target of reducing HCV mortality to less than 2 per 100,000 [4, 5]. However, new targeted case finding methods may be needed to identify individuals who are living with chronic HCV, but as yet undiagnosed or at ongoing risk. Additionally, as two-thirds of people diagnosed with chronic HCV are among those with a past injecting or no injecting risk, these individuals are unlikely to be identified through routine drug service testing [5]. Therefore, new case finding methods will be needed to reach these individuals and to achieve and sustain HCV elimination.

Antenatal screening in the UK routinely offers syphilis, HIV and HBV testing [6], but universal opt-out HCV testing is not currently recommended as part of antenatal screening. This is due to a low risk of mother to child transmission, lack of trial evidence on the efficacy of treatment for HCV in pregnancy, and uncertainty about the prevalence of HCV infection among pregnant women [7]. Opportunistic antenatal HCV testing is currently recommended for women with risk factors for HCV such as being a PWID or being from a high prevalence country [8]. However, there is variation in practice and it has been demonstrated there may be a lack of local guidance on who to test for HCV antenatally [9], despite the national guidance [8], as well as risk-based testing has been found to miss a large proportion (73%) of new diagnoses in London [10].

Given the new era of HCV treatment, where direct-acting antivirals (DAAs) are highly effective and tolerable [11, 12], antenatal testing for HCV may provide a new opportunity to identify new diagnoses among women. There is also the potential to test partners and other children and provide the possibility of re-engagement with care for those who were previously diagnosed but did not initiate or successfully complete treatment. Therefore, the aim of this rapid review was to understand hepatitis C-specific antibody (anti-HCV) and HCV RNA test positivity in antenatal settings in the United Kingdom to inform guidance about HCV antenatal testing. Secondary aims of this rapid review include the uptake and cost-effectiveness of HCV antenatal testing.

## Method

The rapid review was designed and reported following interim guidance from the Cochrane Rapid Reviews Methods Group [13], and the protocol registered at PROSPERO International Register of Systematic Reviews prior to commencing the review (ID CRD42022344265). The PIO (Population, Intervention, Outcome) framework was used to form the search strategy where:

- Population was pregnant women in the United Kingdom.
- Intervention was tested antenatally for HCV.
- Outcome was anti-HCV and HCV RNA test positivity.

Suitable search terms were derived from systematic reviews on similar topics and preliminary searches [14, 15]. Search terms were grouped into two concepts: pregnancy (“antenatal” OR “pregnancy” OR “pregnant” OR “booking bloods”) and hepatitis C (“hepatitis C” OR “HCV” OR “blood borne”), so searches used the string “pregnancy” AND “hepatitis C”. Country specific terms were not added, because preliminary searches revealed that this would exclude suitable articles. The search string was used to search title and abstracts using PubMed (NIH PubMed from 1809 to 30th June 2022) and Web of Science (Web of Science Core Collection from 1900 to 30th June 2022). A period of limit of January 1990 to June 2022 (inclusive) for data collection was imposed to reflect current HCV antenatal testing guidance.

Four stages were used to identify studies: identification, screening, eligibility and inclusion [16]. Title and abstract screening was completed by two reviewers (MH and RS), with a third reviewer used for disagreements (MD). Full-text review, data extraction and quality assessment were completed by one reviewer (MH) with second checking done by a second reviewer (RS). The synthesis without meta-analysis guidance was used for the reporting of results [17]. The critical appraisal tool to assess the quality of cross-sectional studies (AXIS) was used for quality assessment of included studies [18].

Inclusion criteria:

1. Anti-HCV testing reported for pregnant women tested antenatally.
2. Conducted in the United Kingdom.
3. Data collected between January 1990 and June 2022 (inclusive).

Exclusion criteria:

1. Articles not published in English or with no translation available.
2. Studies that focus only on specific sub-populations of pregnant women (e.g. people who inject drugs, migrant women).
3. Qualitative research.

The following data were extracted where available:

- Type of testing method (categorised as universal opt-out or unlinked anonymous testing).
- Uptake of HCV antenatal testing for universal opt-out testing studies.
- The number of women tested for anti-HCV antenatally.
- Anti-HCV and HCV RNA test positivity.
- Cost-effectiveness analyses.

Median values and ranges for anti-HCV positivity and HCV RNA testing are presented as there was too much heterogeneity in studies for meta-analyses to be produced.

## Results

**Fig. 1:**
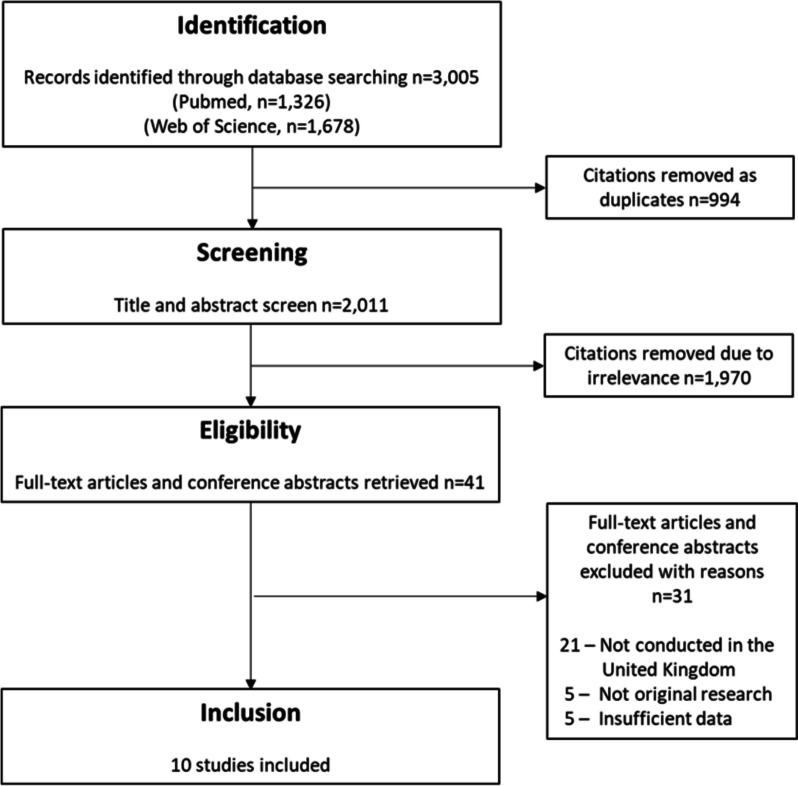
Flow diagram of the identification process

 The searches yielded 2,011 unique citations, of which 1,970 were removed in title and abstract screening and 31 were removed in full-text screening, leaving 10 studies suitable for data extraction and quality assessment (Fig. 1). All studies were cross-sectional, two were conducted in Scotland and eight were conducted in England, with half of all studies conducted in London (*n* = 5). Seven studies used anonymous testing methods (i.e., dried blood spot testing / residual serum sampling) and the remaining three used opt-out testing methods, all of which were conducted in London. A summary of included studies can be seen in Table 1.

**Table 1:** Summary of studies included in the rapid review

Authors	Study design	Study duration	Location	Testing strategy	Uptake N/N (%)	N tested	N anti-HCV positive (%)	N RNA positive (%)	Cost	Quality score (/20)
Ades et al. (2000) [19]	Cross-sectional	April 1997 - June 1998	North Thames, England	Anonymous testing	n/a	126,009	241 (0.19)	n/a	n/a	17
Balogun et al. (2000) [20]	Cross-sectional	January - December 1996	Greater London and the Northern and Yorkshire region, England	Anonymous testing	n/a	London − 25,940 Northern and Yorkshire − 6,675 Total − 32,615	London − 86 (0.33) Northern and Yorkshire − 37 (0.22) Total − 123 (0.37)	London − 55 (0.21) Northern and Yorkshire − 16 (0.24) Total – 71 (0.22)	n/a	17
Carey et al. (2018) [21]	Cross-sectional	August - October 2017	South-East London	Universal opt-out testing	1,012/1,038 (97)	1,012	10 (0.99)	9 (0.89)	Not reported	-
Cortina-Borja et al. (2016) [22]	Cross-sectional	April - June 2012	North Thames, England	Anonymous testing	n/a	31,437	30 (0.10)	n/a	n/a	17
Dannhorn et al. (2015) [23]	Cross-sectional	Unknown	Barking, Havering and Redbridge, London	Anonymous testing	n/a	1000	4 (0.4%)	n/a	n/a	-
Goldberg et al., (2001) [24]	Cross-sectional	January - December 1997	Dundee, Scotland	Anonymous testing	n/a	3,548	23 (0.65)	n/a	n/a	16
Hutchinson et al. (2004) [25]	Cross-sectional	mid March - mid October 2000	Scotland	Anonymous testing	n/a	30,259	88 (0.29)	n/a	n/a	17
Orkin et al. (2016) [26]	Cross-sectional	January - December 2013	London, England	Anonymous testing	n/a	1,000	5 (0.5)	1 (0.1)	n/a	13
Selvapatt et al. (2015) [27]	Cross-sectional	November 2003 - February 2013	London, England	Universal opt-out testing	Not reported	35,355	136 (0.38)	60 (0.17)	£5,469 per newly diagnosed individual Treatment with DAAs - £9,139 per QALY	18
Ward et al. (2000) [28]	Cross-sectional	November 1997 - April 1999	London, England	Universal opt-out testing.	4,729/4,825 (98)	4,729	38 (0.80%)	27 (0.57)	Not reported	18

*QALY* Quality adjusted life years, *DAA *Direct acting antiviral

### Uptake of antenatal HCV testing

Two out of the three studies that used universal opt-out testing methods reported uptake of antenatal HCV testing. The two studies that reported uptake of testing were both based in London and uptake was explored by consent to an HCV antenatal test. The studies found high uptake and reported similar proportions of women accepting HCV antenatal testing (97% and 98%). Neither study explored barriers to uptake of HCV antenatal testing.

### Anti-HCV positivity

All studies reported anti-HCV positivity and test positivity across studies and ranged from 0.1 to 0.99%, with a median value of 0.38% (sample range was 1,000-126,009 women). Range of test positivity for universal opt-out testing was 0.38–0.99% and anonymous testing was 0.1-0.65%. Given the heterogeneity in year and place of study between universal opt-out testing and anonymous testing studies, which will affect anti-HCV test positivity due to differences in prevalence, these cannot be reliably compared.

### HCV RNA testing

Half of the included studies (*n* = 5) included HCV RNA testing. All three studies using universal opt-out testing methods reported RNA testing and two out of seven anonymous testing studies reported RNA testing. In all five studies, 100% of women who were tested anti-HCV positive were subsequently tested for HCV RNA. HCV RNA positivity ranged from 0.1 to 0.57% of the testing population, with a median value of 0.22%. As a proportion of the women testing anti-HCV positive, HCV RNA positivity ranged from 20 to 90% with a median value of 57%. No study investigated risk factors associated with HCV RNA test positivity.

### Cost-effectiveness of antenatal HCV testing

One study presented the cost-effectiveness of antenatal HCV testing based upon maternal outcomes of a screening programme, which was determined to be £5,469 per newly diagnosed individual. The cost of antenatal screening was also presented in terms of quality adjusted life years (QALYs) if treated with DAAs and was calculated to be £9,139 per QALY. This was below national guidelines that consider an intervention cost-effective (£20,000-£30,000 per QALY) [29]. The base case for cost-effectiveness analysis was based on 19 out of 44 women being linked to treatment (43%). Sensitivity analyses revealed increases in underlying HCV prevalence and increases in the proportion linked to treatment yielded better cost-effectiveness, although an HCV prevalence of 0.1% was still deemed cost-effective according to national guidelines.

### Quality assessment

Two studies were not quality assessed due to being conference abstracts. Overall, the quality of studies included was high, with the median score on the AXIS tool being 17 (out of a possible 20). There was one outlier with relatively low scores compared to the other studies (AXIS score = 13). The weakest section for studies tended to be whether information about non-responders was described (*n* = 5), the methods to determine statistical significance were clear (*n* = 3) and whether the limitations were discussed (*n* = 3).

## Discussion

This rapid review found that anti-HCV test positivity among women tested antenatally in the UK ranged from 0.1 to 0.99%, with a median value of 0.38%. HCV RNA test positivity ranged from 0.1 to 0.57% of the testing population, with a median value of 0.22% (among 20–90% of women testing anti-HCV positive, median value = 57%). Reliable comparisons between universal opt-out testing and anonymous testing were not possible due to the differences in the underlying prevalence of HCV in the population between studies. Compared to HBV antenatal testing [30], the median test positivity value was similar (3.77 per 1,000 women tested vs. 3.8 per 1,000 women tested, HBV and HCV respectively) and median number of active HCV, where treatment would be beneficial, was higher among HCV RNA positive women in this review (2.2 per 1,000 women tested) compared to antenatal HBV testing data (0.86 per 1,000 women tested). It therefore appears that testing for HCV antenatally would identify more new diagnoses than testing for HBV.

Previous guidance recommended that antenatal settings perform risk-based testing for HCV, based on factors such as a history of injecting drug use and being born in a high prevalence country [8]. Research has since found that there is variation in how this guidance is implemented [9], and women who do meet the risk criteria can still be missed [10]. Additionally, since the introduction of DAAs, there is a need to re-examine whether providing universal opt-out testing is cost-effective and can improve the quality of life of pregnant women in the UK. Also, a review regarding the role and contribution of HCV antenatal testing in the UK in the context of the UK government’s commitment to WHO elimination goals has not been conducted. This study summarises the current evidence regarding HCV antenatal testing in the UK. In addition to findings relating to test positivity and active HCV in women tested antenatally, this review also aimed to assess cost-effectiveness. One study assessed the cost-effectiveness of HCV antenatal testing in London, which was found to be cost-effective and lower than national thresholds of cost-effectiveness [29]. It is important to note that this study was conducted in London that has a larger migrant population than other regions of the UK and migrant people face a greater burden of HCV than the general population [31]. This may affect the cost-effectiveness of testing in other regions in the UK, but further study is needed. As well as underlying prevalence in the population, cost-effectiveness of HCV antenatal testing is also reliant on services successfully linking individuals to treatment and treatment success [15]. The cost-effectiveness model included was based upon a relatively low proportion of linkage to treatment (43%) and research from England between 2015 and 2019 has found linkage to treatment post antenatal testing was higher than the included study (74%) [32], and therefore likely more cost-effective. Furthermore, the included study was conducted prior to DAA treatment being routinely offered, but also included modelling using DAA treatment costs and benefits, which was also cost-effective. Treatment with DAAs is likely to be more cost-effective than previous interferon treatments, as it is better tolerated and has greater treatment success than previous interferon therapy [11, 12]. Treatment is not currently recommend whilst pregnant or breastfeeding, although longitudinal trials for DAA treatment during pregnancy are currently ongoing [33]. If treatment during pregnancy is determined to be safe to both mother and child, then this may improve cost-effectiveness further, due to immediate treatment availability.

This rapid review attempted to include articles for all of the UK, although most studies were from England and two were from Scotland. Because of the small number of studies from Scotland, it was not deemed suitable to compare findings from the two countries. No studies were found from Northern Ireland and Wales. Given that the different countries that make up the United Kingdom have different overall numbers of HCV infection and different prevalence of HCV [34] and different antenatal care models, different testing strategies (i.e. universal opt-out or targeted screening) may be more suitable depending on the underlying prevalence in each specific country. However, the cost-effectiveness study demonstrated that universal opt-out testing would still be cost-effective at 0.1% and therefore may be suitable across UK nations. Given only one study measured cost-effectiveness of antenatal HCV testing, there may be a need for countries to produce more bespoke cost-effectiveness studies to inform their own HCV testing strategy in the new era of HCV curative treatment and WHO elimination goals.

Two out of the three studies that used universal opt-out testing reported uptake, which was similar for both studies (97% and 98%). This is similar to the national uptake of HBV antenatal testing in England (99.8%) and above the 95% acceptance rate for uptake of HBV antenatal testing [30]. Also, given the relatively small sample size in the included studies relative to the number of women offered antenatal testing (*N* = 662,886), if universal opt out testing was adopted nationally, acceptance of HCV testing should likely be above the 99% achievable threshold [30].

A limitation of this review is that, due to the majority of studies being anonymous testing, only two studies assessed acceptance of HCV opt-out testing in antenatal testing, although this was deemed to be similar to acceptance of HBV antenatal testing [30]. Furthermore, there was too much heterogeneity in studies included to use more advanced methods of data synthesis, such as meta-analyses. To overcome this limitation somewhat, findings were reported according to guidance for synthesis without meta-analysis in review studies [17]. Despite these limitations, the overall quality of evidence included was deemed to be high and this rapid review provides important findings regarding HCV antenatal testing in the UK, which can be used to inform policy.

## Conclusion

Given the UK’s commitment to the WHO targets of eliminating HCV as a public health threat by the year 2030, as well as the new era of HCV treatment where DAAs are highly tolerable and effective, it is important to utilise new methods of case finding and link people living with HCV into treatment. HCV antenatal testing may provide a useful role in new case findings, identifying women who otherwise might not be tested elsewhere. Additionally, it provides an opportunity to test family members and contacts, which would further increase the cost-effectiveness of testing in antenatal settings; this may not only improve an individual’s quality of life but also prevent onward transmission. It is therefore beneficial to reconsider the role universal antenatal opt-out HCV testing may have on helping the UK achieve its HCV elimination targets in the context of a multi-faceted case-finding strategy.

## Data Availability

The datasets used and/or analysed during the current study are available from the corresponding author on reasonable request.

## Abbreviations

DAA: Direct-acting antivirals
HBV: Hepatitis B virus
HCV: Hepatitis C virus
PWID: Person who injects drugs
QALY: Quality adjusted life years
WHO: World health organization
UK: United Kingdom
